# New Design of the Electrophoretic Part of CLARITY Technology for Confocal Light Microscopy of Rat and Human Brains

**DOI:** 10.3390/brainsci9090218

**Published:** 2019-08-29

**Authors:** Petr Zach, Jana Mrzílková, Jan Pala, Libor Uttl, Viera Kútna, Vladimír Musil, Blanka Sommerová, Petr Tůma

**Affiliations:** 1 Department of Anatomy, Third Faculty of Medicine, Charles University, Ruská 87, 100 00 Prague 10, Czech Republic; 2 National Institute of Mental Health, Topolová 748, 250 67 Klecany, Czech Republic; 3 Department of Pathophysiology, Third Faculty of Medicine, Charles University, Ruská 87, 100 00 Prague 10, Czech Republic; 4 Centre of Scientific Information, Third Faculty of Medicine, Charles University, Ruská 87, 100 00 Prague 10, Czech Republic; 5 Department of Hygiene, Third Faculty of Medicine, Charles University, Ruská 87, 100 00 Prague 10, Czech Republic

**Keywords:** brain, CLARITY, electrophoresis, microscopy, instrumentation

## Abstract

Background: CLARITY is a method of rendering postmortem brain tissue transparent using acrylamide-based hydrogels so that this tissue could be further used for immunohistochemistry, molecular biology, or gross anatomical studies. Published papers using the CLARITY method have included studies on human brains suffering from Alzheimer’s disease using mouse spinal cords as animal models for multiple sclerosis. Methods: We modified the original design of the Chung CLARITY system by altering the electrophoretic flow-through cell, the shape of the platinum electrophoresis electrodes and their positions, as well as the cooling and recirculation system, so that it provided a greater effect and can be used in any laboratory. Results: The adapted CLARITY system is assembled from basic laboratory components, in contrast to the original design. The modified CLARITY system was tested both on rat brain stained with a rabbit polyclonal anti-Iba-1 for microglial cells and on human nucleus accumbens stained with parvalbumin and tyrosine hydroxylase for visualization of specific neurons by confocal laser scanning microscopy. Conclusions: Our design has the advantage of simplicity, functional robustness, and minimal requirement for specialized additional items for the construction of the CLARITY apparatus.

## 1. Introduction

CLARITY is a technique used to transform intact brain tissue into an optically transparent and permeable hydrogel form that can then be used for immunostaining and high-resolution 3-D imaging without damage to the sample [1,2]. This also includes imaging of neural projections, local circuit wiring, cellular relationships, subcellular structures, protein complexes, nucleic acids, and neurotransmitters [1]. The CLARITY principle consists of the following steps: (i) an extensive area of the brain with an edge of up to several centimeters is penetrated with acrylamide; (ii) after the acrylamide penetrates the entire 3-D structure, polymerization proceeds with the formation of a hydrogel; (iii) then the hydrogel is washed with a solution of SDS to remove lipids and is thus cleared for 3-D imaging techniques; (iv) removal of the lipids by washing the hydrogel with a stream of an SDS solution is time-consuming and can be substantially accelerated by simultaneous application of electrophoresis, where the hydrogel is placed in a direct current (DC) electric field and the incorporation of SDS micelles into the hydrogel is controlled electrophoretically; (v) finally, coloring is performed for a microscopic imaging technique, such as in situ hybridization or antibody labeling [3,4].

The advantages of CLARITY include the preservation of tissue morphology and subcellular structures [5]. Disadvantages consist in the multiple-step process that takes place over several days/weeks, time-consuming immunostaining for thicker tissue samples and high start-up and consumable costs [3]. A number of attempts have been made to modify the original CLARITY protocol [6,7,8], including rotation of the electrophoretic flow-through cell, optimization of brain sample imaging or passive CLARITY as the most important. Recently, the combination of expansion microscopy and lattice light-sheet microscopy with high imaging speed has been used [9], low photobleaching, and 3D nanoscale resolution below 70 nm generated images of subcellular structures of Drosophila fly brain and mouse brain cortical tissue.

Chemically, the performance of the CLARITY technique is quite simple, consisting of the following prescribed procedures, as all the required chemicals are normally available. However, problems are encountered in the instrumental part of the CLARITY technique, required for washing lipids out from the hydrogel formed inside the tissue, rendering the whole area transparent for microscopy. Suitable instrumentation is not commercially available and thus it must be assembled in the laboratory. This consists of the construction of a flow-through electrophoretic cell and assembling of the hydrodynamic and cooling equipment. This communication provides a detailed description of an alternative construction of the flow-through electrophoretic cell and a design for the complete washing equipment. The cell was constructed from common materials and means available from laboratory equipment suppliers and the other equipment can be purchased from normal retail outlets. The new design is characterized by a simple design, functional robustness, and minimal requirements on additional equipment. A complete functional prototype of the flow-through electrophoretic cell can be readily constructed in the laboratory using the procedure described below. In addition, we also focused on designing an optimal protocol for subsequent confocal microscopy using this modified CLARITY protocol.

## 2. Materials and Methods

### 2.1. Construction of a Flow-Through Electrophoretic Cell

**Figure 1:**
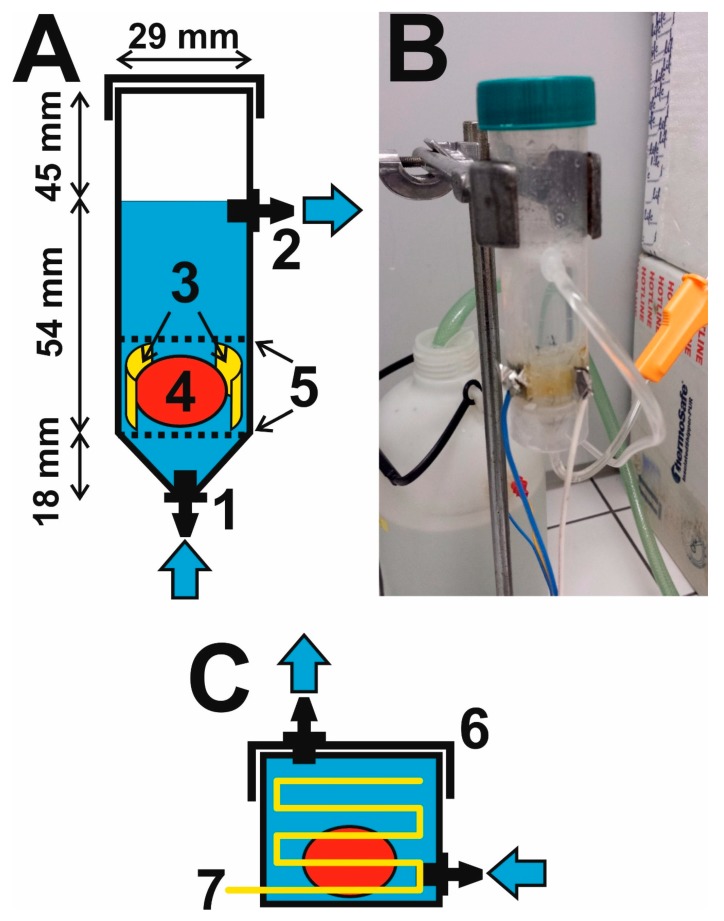
Scheme of the electrophoretic flow-through cell made from the polyethylene (PE) vessel (**A**): 1—inlet and 2—outlet small fitting for connecting tubing, 3—Pt-electrodes, 4—hydrogel tissue, 5—plastic meshes; (**B**) photograph of the cell; (**C**) original flow-through cell of CLARITY apparatus for comparison: 6—PE vessel with flat bottom, 7—Pt-electrodes created from rectangular-shaped wires.

**Figure 2:**
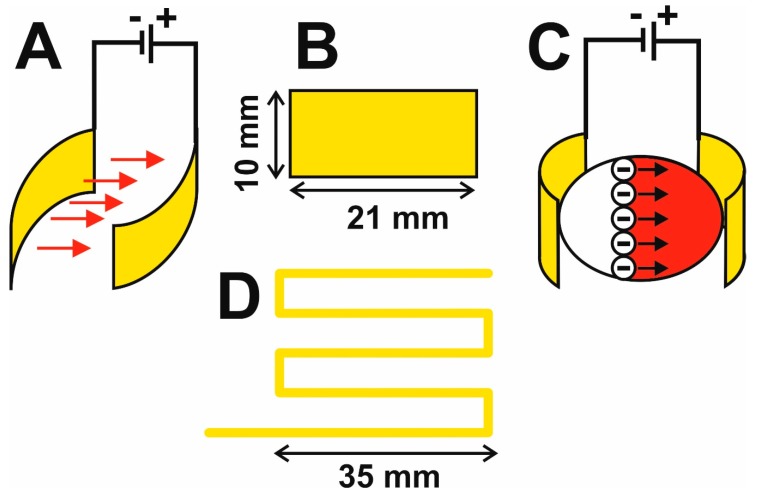
Arrangement of the flat Pt-electrodes inside the cell (**A**); dimensions of the electrodes (**B**); direction of the electrophoretic washing out of the lipids with SDS micelles (**C**); Pt-wire-electrode of the original CLARITY apparatus for comparison (**D**).

The electrophoretic cell was made from a 50 mL polyethylene (PE) vessel with a conical base and screw cap, which is normally used for collecting urine (P-LAB, Prague, Czechia). The sampling vessel was modified for use in the flow-through cell as follows (Figure 1):

(i) Two holes with a diameter of 4 mm were drilled into the top of the conical base and the vertical wall cca 45 mm from the upper edge of the vessel. Plastic connectors for connecting rubes, a small Luer fitting for 1/8” tubing (the Luer valve assortment set, from World Precision Instruments, Saratosa, FL, USA, was used), were forced into the openings; the drilled hole must be slightly smaller than the diameter of the connector so that a water-tight connection is formed after pressing it in. The connectors were joined to transparent PE extension tubing for infusion probes with a length of cca 70 cm (Extension line, Braun, Melsungen, Germany). The other end of the lower tube is connected to the outlet stopcock of a 5 L stock bottle (P-LAB), once again using a fitting (World Precision Instruments, Saratosa, FL, USA), and is used to bring the washing solution to the cell. The other end of the upper tubing is loosely inserted into an opening in the screw top of a second 5 L stock bottle, which is used to collect solution flowing out of the cell.

(ii) The electrophoretic part of the cell consists of two platinum foils with a length of cca 2.1 cm, height of 1.0 cm, and thickness of 0.2 mm (Safina, Vestec, Czechia), acting as electrodes. The Pt-foils are inserted longitudinally into vertical slits cut with a scalpel in the lower part of the vessel (there is a space of cca 10 mm between the slits) so that 2 mm of the foil remains outside the vessel. Each slit is thoroughly sealed around its circumference with a special adhesive for PE (Ceys, AC Marca, Český Brod, Czechia), forming a mechanically strong and water-tight connection between the electrode and the outer surface of the vessel. Insulated copper wires are then soldered to the outside parts of the Pt-electrodes for connecting the DC voltage, see Figure 2.

(iii) The hydrogel tissue is fixed inside the vessel using PE mesh with a hole size of 1–2 mm. The material for the mesh was window netting against insects, from which two circular pieces were cut; the first formed the bottom, on which the hydrogel tissue is placed, and the second is above the hydrogel and it is fixed in a position between the flat Pt-electrodes. It is important to use the meshes to keep the hydrogel between the electrodes at a place with high electric field intensity; otherwise, the hydrogel moves freely in the vessel under the effect of turbulent flow of the solution, substantially reducing the effectiveness of the electrophoretic washing and the hydrogel can be damaged mechanically.

### 2.2. Putting Together the Electrophoretic Washing Equipment

**Figure 3:**
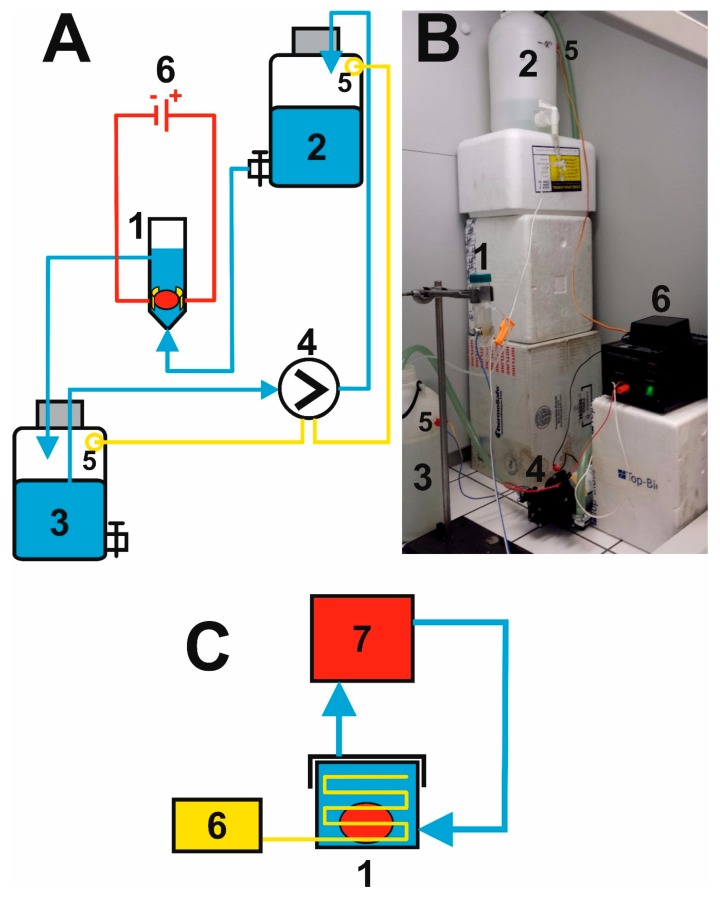
Scheme of the flow-through system (**A**): 1—vessel with the hydrogel, 2—upper and 3—lower bottle of washing solution, 4—membrane pump, 5—sensor, 6—source of direct current (DC) voltage; (**B**) photograph of the whole arrangement; (**C**) scheme of the original CLARITY apparatus for comparison: 1—vessel with the hydrogel, 6—source of DC voltage, 7—circulator of clearing solution including pumping and cooling system.

The electrophoretic flow-through cell is fixed in place using metal clamps and a chemical stand about 50 cm above the work surface of the fume cupboard. The input opening at the bottom of the cell is connected to PE tubing, bringing in the washing solution from the stock bottle located at a height of cca 100 cm above the work surface (Figure 3). The washing solution flows through the cell under the force of gravitation from the bottom upwards at an average flow rate of 1 L/min, washes the surface of the hydrogel and flows out of the cell through the upper opening, from which it is fed through the other PE tubing to the lower stock bottle standing on the work surface. The solution is pumped from the lower to the upper vessel by a diaphragm pump (LS403, LILIE GmbH & Co. KG, Besigheim, Germany, pressure 2.1 bar, flow rate 10.6 L/min, 12 V charging voltage), originally designed for a garden irrigation system, which pumps the entire volume of 5 L in 0.5 min; the pump is connected to a 1/2” irrigation hose passing through the lids of the two vessels. Transfer of the washing solution is controlled automatically by two contact sensors located 5 cm under the upper edge of the stock bottles and proceeds as follows: When the surface of the solution in the bottom bottle reaches the position of the sensor, a switching relay is turned on and the pump begins to pump solution into the upper bottle. The pumping stops when the solution in the upper bottle reaches the position of the sensor, activating the switching relay and the pump is turned off. The whole process proceeds sequentially and was operated without any defect for a test period of 5 days.

The washing out of lipids from the hydrogel is accelerated by applying a DC voltage to the Pt-electrodes (DC Power Supply KXN-2005D, Zhaoxin, Shanghai, China). At an optimum value of the working voltage of 50 V, a current of about 1.1 A flows through the circuit and the temperature of the solution inside the cell does not exceed 42 °C, so that the hydrogel is not thermally degraded. The washing solution is electrolyzed at the Pt-electrodes, manifested in the evolution of bubbles of oxygen at the anode and of hydrogen at the cathode.

### 2.3. Tissue Samples, Fixation, and Preparation

**Figure 4:**
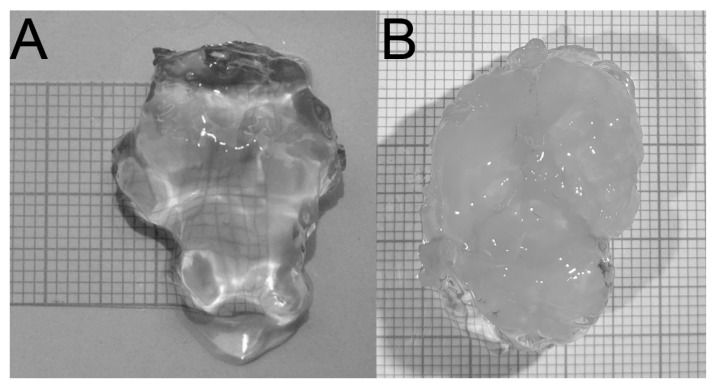
Comparison of CLARITY processed rat brains after 24 hours. (**A**) Transparent hydrogel of rat brain by adapted CLARITY apparatus. (**B**) Original designed CLARITY apparatus (courtesy of Charles University, Prague, Czech Republic). The millimeter grids are attached in the background.

Rat brains were obtained from adult male Wistar rats (10 months old) from the breeding colony (The Institute of Physiology, Academy of Sciences of CR). The rats were anaesthetized using 5% *m*/*v* pentobarbital intraperitoneally and perfused with standard physiological solution, then decapitated and their brains were extracted from their skulls and placed in a physiological solution for 24 hours. The cerebellum and brainstem were separated from the rest of the brain. The remaining brain tissue (part of telencephalon and diencephalon) was processed using the CLARITY system to form a transparent hydrogel, see Figure 4. Human brains (4 males, aged 70–72 years, postmortem interval 4–6 days, without brain fixation in paraformaldehyde) were extracted from the skull of post mortem cadavers and dissected under a light magnifier (Fisher Scientific, Loughborough, UK). Nucleus accumbens was dissected from a coronal brain section as a cubic block specimen (5 × 5 mm) and treated by the CLARITY procedure.

### 2.4. Preparation of Solutions for CLARITY

All the chemicals were of analytical grade purity and all the solutions were prepared using deionized Milli-Q water (DEI, 18.2 MΩ cm, Millipore, Molsheim, France).

#### 2.4.1. Components for Hydrogel Preparation

(i) the 40% *m*/*v* acrylamide (Amersham Biosciences, Uppsala, Sweden) solution was prepared in DEI and stored at 4 °C; (ii) 2% *m*/*v* bis-acrylamide (N,N-methylenebisacrylamide, Amersham Biosciences, Uppsala, Sweden) was prepared in DEI and stored at 4 °C; (iii) the phosphate-buffered saline stock solution (stock PBS) was prepared by dissolving 8.0 g NaCl (Lachema, Brno, Czechia), 0.2 g KCl (Lachema, Brno, Czechia), 1.44 g Na_2_HPO_4_ (Sigma-Aldrich, Steinheim, Germany), and 0.24 g KH_2_PO_4_ (Sigma-Aldrich, Steinheim, Germany) in 100 mL of DEI and the pH of the solutions was adjusted to 7.4 by 1 M HCl; iv) the 10% *m*/*v* paraformaldehyde (Lachema, Brno, Czechia) solution was dissolved in hot DEI and stored at 4 °C.

#### 2.4.2. Penetration of Brain Tissue by the Hydrogel Solution

125 mg of VA-044 Initiator of polymerization (2,2-azobis (2-(2-imidazolin-2-yl) propane) dihydrochloride, Waco Chemicals, Neuss, Germany) was weighed into a 50 mL polyethylene tube and the following were added stepwise: 5 mL 40% *m*/*v* acrylamide solution, 1.25 mL 2% *m*/*v* bis-acrylamide solution, 5 mL 10 fold diluted stock PBS, and 20 mL 10% *m*/*v* paraformaldehyde solution. After mixing, the brain sample was immersed in the hydrogel solution and cold DEI was added to a final volume of 50 mL. The brain samples were incubated in the hydrogel solution at 4 °C for 2–3 weeks.

#### 2.4.3. Hydrogel Tissue Embedding

Initially, one half of the hydrogel solution was removed before the acrylamide polymerization, which was performed in the same 50 mL PE flask used for sample penetration. First, the lid of the flask was replaced with a modified one equipped with a 4 mm hole and a male Luer fitting for attaching a laboratory vacuum pump (Julabo Laboport, Seelbach, Germany). The hydrogel solution was degassed under a vacuum of 0.9 bars (90 kPa) for 10 min. Following this procedure, the lid of the 50 mL flask was replaced with a common screw lid and the flask was placed in a water bath (Julabo TWB 5, Julabo Labortechnik, Seelbach, Germany) and incubated at 37 °C for 3 hours. Finally, the polymerized sample was removed from the flask, the excess gel was removed from the sample surface and the treated sample was inserted into an electrophoretic flow-through cell.

#### 2.4.4. Preparation of the Clearing Solution

The clearing solution was prepared by dissolving: (i) 200 g of sodium dodecyl sulfate (SDS, Sigma-Aldrich, Steinheim, Germany) in 1.0 L of warm DEI and (ii) 62 g of boric acid (Sigma-Aldrich, Steinheim, Germany) in 4.0 L of DEI and the pH was adjusted to 8.5 with solid NaOH (Penta, Prague, Czechia). The two solutions were mixed in a storage bottle and used for electrophoretic clearing experiments.

### 2.5. Brain Tissue Staining and Confocal Microscopy

#### 2.5.1. Brain Tissue Staining for Confocal Microscopy

CLARITY-processed brains were cut using a rat brain slicer (Adult Rat Brain Slicer Matrix BSRAS002-1, Pittsburgh, PA, USA) to 2 mm thick coronal slices. The brain slices were then washed with 0.1 M PBS, pH 7.4, for 24 hours. The specimens were incubated with primary antibody diluted in 0.1 M PBS and 0.3% *m*/*v* Triton-X with the addition of 0.01% *m*/*v* sodium azide for 7 days on an orbital shaker at room temperature (RT). The primary antibody was a rabbit polyclonal anti-Iba-1 (Wako, Osaka, Japan, 1:100) for rat brain. Immunohistochemistry of humans brain was carried out using 100 fold dilution of rabbit polyclonal anti-tyrosine hydroxylase antibody (TH, Millipore, Temecula, CA, USA, 1:100) and mouse monoclonal anti-parvalbumin antibody (PV, Sigma, St. Louis, MO, USA, 1:100). After incubation with the primary antibody, the sections were washed with 0.1 M PBS for 24 hours. Tissue slices were incubated with AlexaFluor 488 conjugated donkey anti-rabbit secondary antibody (Jackson ImmunoResearch, Baltimore, MD, USA) and AlexaFluor 594 conjugated donkey anti-mouse secondary antibody (Jackson ImmunoResearch, Baltimore, MD, USA).. The secondary antibodies were 100 fold diluted in 0.1 M PBS and 0.3% *m*/*v* Triton-X and incubated on an orbital shaker at RT for 7 days. The tissue was then washed with 0.1 M PBS for 24 hours at RT to remove excess unbonded antibody.

#### 2.5.2. Confocal Microscopy Procedure

**Figure 5:**
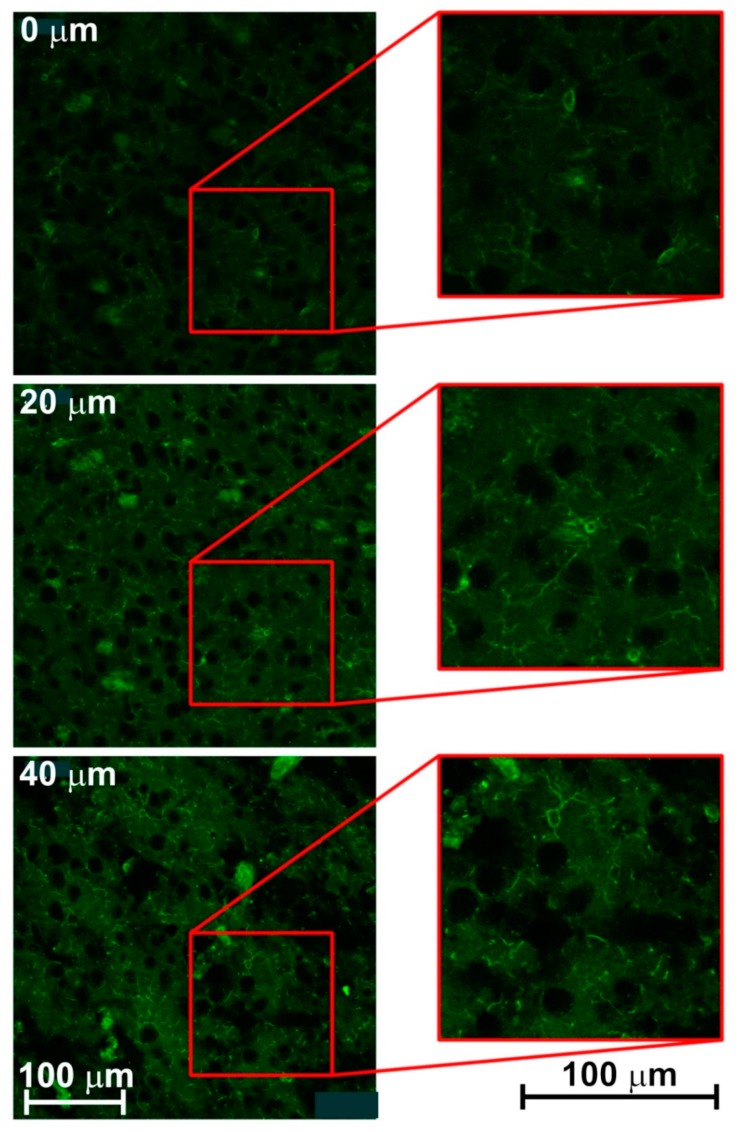
Images of the rat brain parietal cortex created by one-photon confocal laser scanning microscope Leica TCS SP8 X after hydrogel-forming and staining; experimental conditions in 2.5 Brain tissue staining and confocal microscopy. Three different samples at depths 0, 20, and 40 μm of histological sections of the parietal cortex of rat brain employing Iba-1 staining. Positive microglial cells appear green.

**Figure 6:**
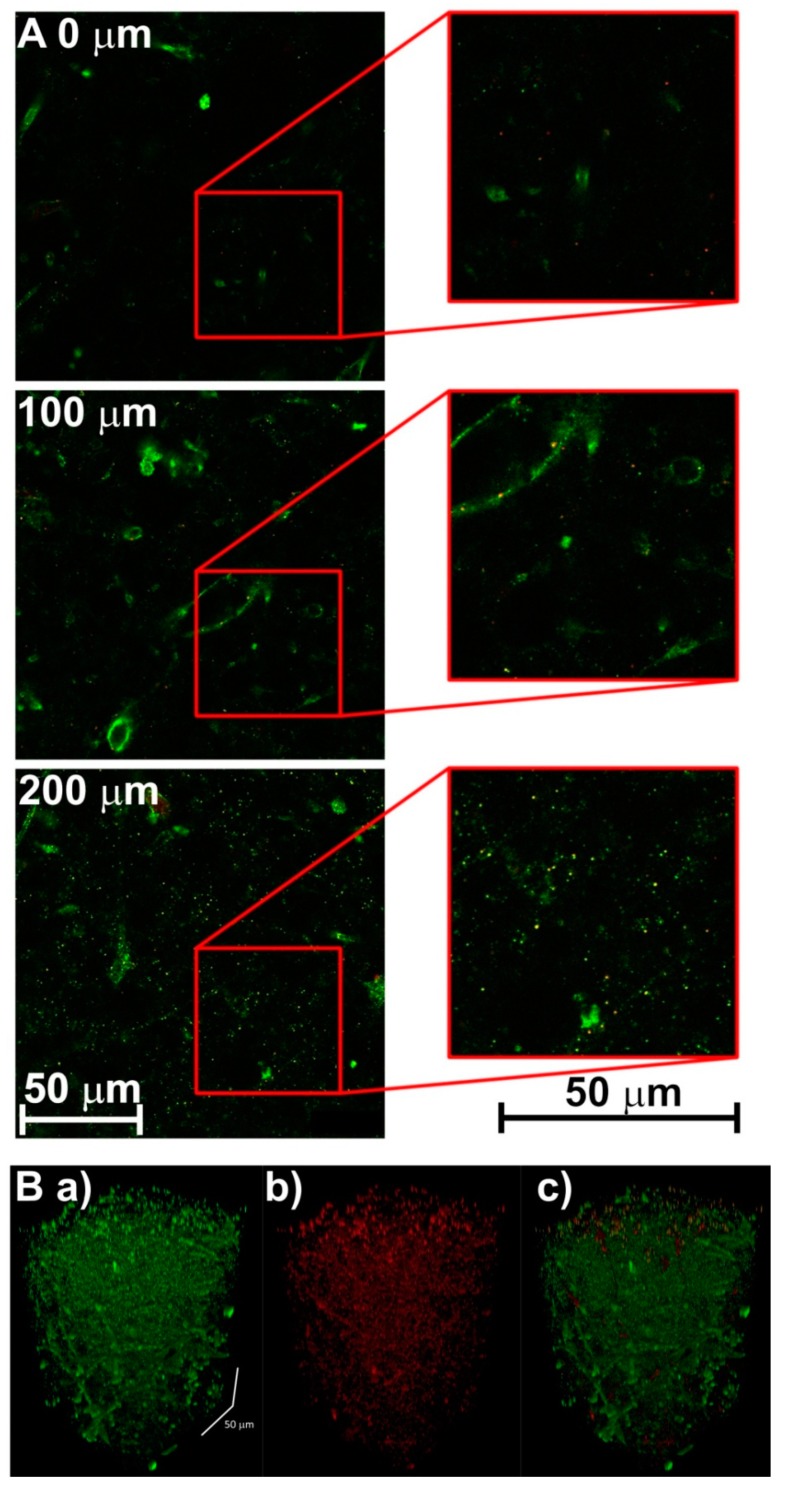
Images of the human nucleus accumbens created by one-photon confocal laser scanning microscope Leica TCS SP8 X after hydrogel-forming and staining; experimental conditions in 2.5 Brain tissue staining and confocal microscopy. Histological sections at different depths of 0, 100, and 200 µm of human nucleus accumbens after immunohistological staining (**A**). Parvalbumin positive fibers of gamma-aminobutyric acid (GABA) neurons appear green and tyrosine hydroxylase stained dopamine neurons appear red. 3-D reconstruction of brain tissue (**B**) performed to a depth of 230 µm with a depiction of the bodies of individual neurons: (**a**) GABA neurons, (**b**) dopamine neurons, (**c**) both types of neurons together.

Labelled brain slices were placed in a Cellvis Petri dish with 20 mm round bottom coverslips (^#^ 1.5) and supported by 0.1 M PBS. Microscopic imaging of the samples was performed with a high-end confocal laser scanning microscope Leica TCS SP8 X equipped with a white light laser (emitting in the spectral range 470–670 nm) and a near UV laser 405 nm, and two freely tunable spectral detectors: A PhotoMultiPlier and a hybrid detectors based on Gallium Arsenide Phosphide (GaAsP), see (Figure 5 and Figure 6). Leica objectives Plan Apo 10 ×/0.40 dry (working distance 2.2 mm) for low-resolution and Plan Apo 40/1, 10 Water (working distance 0.65 mm) for high-resolution were also used (Figure 5 and Figure 6). The attenuation of excitation laser power (and the associated emitted fluorescence signal) with increasing penetration depth was compensated by Acoustical Optical Tunable Filter settings of excitation lasers. 

### 2.6. Ethical and Legal Statement

Human brains were obtained, on the basis of the informed consent from all the individual participants included in the study, for the Donor Program to the Third Faculty of Medicine, Charles University, Prague, CR and the study of human brains was approved by the Ethical Committee of the Third Faculty of Medicine (No. 12/2016). All the experimental procedures were approved by the Expert Committee for Protection of Experimental Animals of the Third Faculty of Medicine and were performed in accordance with the Animal Protection Act of the Czech Republic and respected the Guidelines of the Council of the European Union (86/609/EU).

## 3. Results and Discussion

### 3.1. Innovation of the Electrophoretic Washing Cell

Compared to the original design, the described electrophoretic washing cell has a number of innovative features: (i) the wire electrodes are replaced by flat electrodes, which are shaped so that they closely envelop the hydrogel. This forms a homogeneous high-intensity electric field which is focused directly to the center of the vessel. The electrophoretic washing out of the lipids progresses evenly over the whole area gradually from the cathode to the anode and negatively charged SDS micelles enter the hydrogel, Figure 2C. Under these conditions, the piece of rat brain hydrogel with a length of over 20 mm is cleared in only 24 hours, which is substantially faster than the several days required for the original construction with wire electrodes; the most time-consuming step of the CLARITY process is reduced significantly, see Figure 4. The short washing time simultaneously improves the quality of the obtained hydrogel for subsequent microscopic examination, as long soaking of the hydrogel in the washing solution leads to its decomposition. (ii) The second aspect is the hydrodynamics of the employed design. The flow of the washing solution is controlled by gravitation, where the solution flows from the upper stock vessel through the electrophoretic cell to the lower vessel. The pumping of the solution from the lower to the upper stock vessel occurs only in impulses and is performed by an ordinary garden pump. This eliminates the need for continuous pumping of the washing solution and prolongs the lifetime and reliability of the whole system. (iii) The washing solution flows through thin tubing and is effectively cooled by the surrounding air; during continuous operation, the temperature of the solution increased to a maximum of 42 °C, where the laboratory temperature was 25 °C. If required, the lower vessel can be placed in a thermostat.

The overall quality of the electrophoretic washing cell is documented by obtaining completely transparent rat brain hydrogel with a longest dimension of 27 mm, Figure 4. A sample prepared in this way was used for microscopic examination [10]. All innovations and advantages of the described new CLARITY design are clearly compared with the original CLARITY apparatus and highlighted in Figure 1, Figure 2, Figure 3 and Figure 4.

### 3.2. Creating 3-D Images of Brain Tissue

Specimens processed by CLARITY can be visualized using several different microscopic imaging techniques. The most convenient of these image acquisition techniques for any kind of deep tissue imaging including CLARITY is multiphoton microscopy with excitation in the infrared part of the spectrum (700–1080 nm) using a special infrared objective with long working distance and high numerical aperture. Here, a standard one-photon confocal laser scanning microscopy equipped with deconvolution, post-processing and stimulated emission depletion was used; the achieved resolution is approximately 100 nm in the *XY* plane [11]. In comparison to multiphoton microscopy, our approach based on standard one-photon confocal microscopy and standard objectives has some drawbacks, such as lower penetration depth, lower signal to noise ratio, greater photobleaching, and greater scattering of the emitted fluorescence in the brain tissue [8].

Mouse parietal cortex of the brain sections was stained for Iba-1 positive microglial cells (Figure 5) and human nucleus accumbens sections were stained for parvalbumin-positive gamma-aminobutyric acid (GABA) neurons and tyrosine hydroxylase dopaminergic neurons (Figure 6). We selected staining for parvalbumin-positive GABA neurons and tyrosine hydroxylase dopaminergic neurons because of its relevance for neuropsychiatric clinical studies. For example, differences in the distribution of tyrosine hydroxylase immunolabeling in the nucleus accumbens in the post mortem brains of patients with schizophrenia and healthy controls have been reported [12]. In addition, an increase in Iba-1 expression in microglial cells was observed in cases of brain ischemia, injury, and neuroinflammation [13,14,15].

The recently developed CLARITY technique has great potential for the 3-D spatial reconstruction of brain tissue or even whole brains (e.g., small animals) and can, therefore, be employed in studying neurodegenerative diseases as a suitable companion to neuroanatomical correlates [2,16]. For this reason, demand is increasing for the implementation of CLARITY in a wide range of research facilities worldwide. From the point of view of chemistry, CLARITY represents a simple technique relying on published laboratory protocols and generally available laboratory equipment. A minor challenge could be encountered during the procedure when it is necessary to wash out lipids from the hydrogel formed inside the sample tissue. This step leads to specimen transparency with a further possibility of 3-D visualization by various optical microscopes. Nevertheless, since there is no commercially available hardware, individual laboratories will need to design and create the electrophoretic apparatus for CLARITY applications. The critical aspect here is the construction of the electrophoretic flow-through cell and the technical aspects of the hydrodynamic and electrophoretic arrangement of the apparatus. Our article offers a detailed description of an alternative method for designing and constructing the electrophoretic flow-through cell and a blueprint for the complete wash-out apparatus. The materials used in our design can be readily obtained from laboratory equipment suppliers. Our design describes a simple, functional, and easily assembled apparatus for use with the CLARITY protocol with minimal requirements for specialized items.

## 4. Conclusions

The innovative design of the electrophoretic flow-through cell substantially accelerates the process of clearing the hydrogel and so it reduces the negative impact of electric current and the whole washing procedure on the quality of brain tissue for subsequent imaging techniques. The modifications include the size, shape, and arrangement of the Pt-electrodes, the passive cooling of the washing solution by the surrounding air and sequence pumping of the washing solution without the necessity of using special equipment. The proposed arrangement is characterized by simplicity and the detailed description of the design, including the employed materials and auxiliary equipment, provides a number of laboratory suggestions for the practical implementation of the CLARITY technique. We believe that this procedure can provide many laboratories with a manual for the practical implementation of the CLARITY method.

## References

[1] Chung K., Wallace J., Kim S.Y., Kalyanasundaram S., Andalman A.S., Davidson T.J., Mirzabekov J.J., Zalocusky K.A., Mattis J., Denisin A.K. (2013). Structural and molecular interrogation of intact biological systems. Nature.

[2] Chung K., Deisseroth K. (2013). Clarity for mapping the nervous system. Nat. Methods.

[3] Tomer R., Ye L., Hsueh B., Deisseroth K. (2014). Advanced clarity for rapid and high-resolution imaging of intact tissues. Nat. Protoc..

[4] Epp J.R., Niibori Y., Hsiang H.L., Mercaldo V., Deisseroth K., Josselyn S.A., Frankland P.W. (2015). Optimization of clarity for clearing whole-brain and other intact organs. eNeuro.

[5] Liu A.K.L., Hurry M.E.D., Ng O.T.W., DeFelice J., Lai H.M., Pearce R.K.B., Wong G.T.C., Chang R.C.C., Gentleman S.M. (2016). Bringing clarity to the human brain: Visualization of lewy pathology in three dimensions. Neuropathol. Appl. Neurobiol..

[6] Kim S.Y., Cho J.H., Murray E., Bakh N., Choi H., Ohn K., Ruelas L., Hubbert A., McCue M., Vassallo S.L. (2015). Stochastic electrotransport selectively enhances the transport of highly electromobile molecules. Proc. Natl. Acad. Sci. USA.

[7] Poguzhelskaya E., Artamonov D., Bolshakova A., Vlasova O., Bezprozvanny I. (2014). Simplified method to perform clarity imaging. Mol. Neurodegener..

[8] Phillips J., Laude A., Lightowlers R., Morris C.M., Turnbull D.M., Lax N.Z. (2016). Development of passive clarity and immunofluorescent labelling of multiple proteins in human cerebellum: Understanding mechanisms of neurodegeneration in mitochondrial disease. Sci. Rep..

[9] Gao R.X., Asano S.M., Upadhyayula S., Pisarev I., Milkie D.E., Liu T.L., Singh V., Graves A., Huynh G.H., Zhao Y.X. (2019). Cortical column and whole-brain imaging with molecular contrast and nanoscale resolution. Science.

[10] Woo J., Lee M., Seo J.M., Park H.S., Cho Y.E. (2016). Optimization of the optical transparency of rodent tissues by modified pact-based passive clearing. Exp. Mol. Med..

[11] Unnersjo-Jess D., Scott L., Blom H., Brismar H. (2016). Super-resolution stimulated emission depletion imaging of slit diaphragm proteins in optically cleared kidney tissue. Kidney Int..

[12] McCollum L.A., McCullumsmith R.E., Roberts R.C. (2016). Tyrosine hydroxylase localization in the nucleus accumbens in schizophrenia. Brain Struct. Funct..

[13] Ito D., Tanaka K., Suzuki S., Dembo T., Fukuuchi Y. (2001). Enhanced expression of iba1, ionized calcium-binding adapter molecule 1, after transient focal cerebral ischemia in rat brain. Stroke.

[14] Donat C.K., Scott G., Gentleman S.M., Sastre M. (2017). Microglial activation in traumatic brain injury. Front. Aging Neurosci..

[15] Jeong H.K., Ji K., Min K., Joe E.H. (2013). Brain inflammation and microglia: Facts and misconceptions. Exp. Neurobiol..

[16] Lee E., Choi J., Jo Y., Kim J.Y., Jang Y.J., Lee H.M., Kim S.Y., Lee H.J., Cho K., Jung N. (2016). Act-presto: Rapid and consistent tissue clearing and labeling method for 3-dimensional (3d) imaging. Sci. Rep..

